# Technology Usability for People Living With Dementia: Concept Analysis

**DOI:** 10.2196/51987

**Published:** 2024-07-03

**Authors:** Shao-Yun Chien, Oleg Zaslavsky, Clara Berridge

**Affiliations:** 1 School of Nursing, University of Washington Seattle, WA United States; 2 School of Social Work, University of Washington Seattle, WA United States

**Keywords:** usability, dementia, older adults, technology, concept analysis, mobile phone

## Abstract

**Background:**

Usability is a key indicator of the quality of technology products. In tandem with technological advancements, potential use by individuals with dementia is increasing. However, defining the usability of technology for individuals with dementia remains an ongoing challenge. The diverse and progressive nature of dementia adds complexity to the creation of universal usability criteria, highlighting the need for focused deliberations. Technological interventions offer potential benefits for people living with dementia and caregivers. Amid COVID-19, technology’s role in health care access is growing, especially among older adults. Enabling the diverse population of people living with dementia to enjoy the benefits of technologies requires particular attention to their needs, desires, capabilities, and vulnerabilities to potential harm from technologies. Successful technological interventions for dementia require meticulous consideration of technology usability.

**Objective:**

This concept analysis aims to examine the usability of technology in the context of individuals living with dementia to establish a clear definition for usability within this specific demographic.

**Methods:**

The framework by Walker and Avant was used to guide this concept analysis. We conducted a literature review spanning 1984 to 2024, exploring technology usability for people with dementia through the PubMed, Web of Science, and Google Scholar databases using the keywords “technology usability” and “dementia.” We also incorporated clinical definitions and integrated interview data from 29 dyads comprising individuals with mild Alzheimer dementia and their respective care partners, resulting in a total of 58 older adults. This approach aimed to offer a more comprehensive portrayal of the usability needs of individuals living with dementia, emphasizing practical application.

**Results:**

The evidence from the literature review unveiled that usability encompasses attributes such as acceptable learnability, efficiency, and satisfaction. The clinical perspective on dementia stages, subtypes, and symptoms underscores the importance of tailored technology usability assessment. Feedback from 29 dyads also emphasized the value of simplicity, clear navigation, age-sensitive design, personalized features, and audio support. Thus, design should prioritize personalized assistance for individuals living with dementia, moving away from standardized technological approaches. Synthesized from various sources, the defined usability attributes for individuals living with dementia not only encompass the general usability properties of effectiveness, efficiency, and satisfaction but also include other key factors: adaptability, personalization, intuitiveness, and simplicity, to ensure that technology is supportive and yields tangible benefits for this demographic.

**Conclusions:**

Usability is crucial for people living with dementia when designing technological interventions. It necessitates an understanding of user characteristics, dementia stages, symptoms, needs, and tasks, as well as consideration of varied physical requirements, potential sensory loss, and age-related changes. Disease progression requires adapting to evolving symptoms. Recommendations include versatile, multifunctional technology designs; accommodating diverse needs; and adjusting software functionalities for personalization. Product feature classification can be flexible based on user conditions.

## Introduction

### Improving Technology Usability for Dementia

Usability, a critical determinant of technology’s quality, influences user acceptance and overall experience, with its importance magnified for individuals with mild cognitive impairment or dementia [1-3]. The intricate nature of modern technologies necessitates designs that accommodate cognitive limitations, ensuring accessibility and ease of use for this demographic. However, the specific usability needs of those with dementia are often not adequately defined given the condition’s variability and progression [4]. This underdefinition underscores the necessity for technology products to be designed with a deep understanding of the cognitive, memory, and learning challenges faced by individuals with dementia. It is critical to ensure that technology is designed not only to be convenient but also to address specific needs and enhance usability across the spectrum of dementia types, thereby facilitating its adoption and effective use.

Worldwide, >50 million people are living with dementia, a number projected to triple by 2050 [5,6]. Every 3 seconds, someone is diagnosed with dementia, posing significant challenges to health care, care provision, and social services worldwide [7]. Technological interventions, including digital tools such as calendars and talking watches, assistive devices, and telecare, offer potential benefits by aiding daily function, improving safety, and enhancing social connectedness, thereby improving life quality and mood [8-11]. Moreover, these interventions can alleviate caregiver burden by providing monitoring and reminders, with a potential to supplement human care [8,12]. The COVID-19 pandemic has further highlighted the crucial role of technology in health care, emphasizing its importance in maintaining quality care [13]. In alignment with the World Health Organization’s “Dementia: a public health priority” report, advancements in communication and assistive technologies have provided a variety of intervention methods to improve the lives and health of people living with dementia, including maintaining independent living and enhancing safety and autonomy [14].

However, technological solutions must be designed with the specific needs and abilities of people living with dementia in mind to avoid confusion, frustration, and potential rejection due to complexity or unintuitive interfaces [15,16]. The World Health Organization’s 2022 policy brief on agism in artificial intelligence (AI) technologies underscores the importance of addressing stereotypes, biases, or discrimination in AI, ensuring quality care for older adults [17]. Tailoring AI technology to the diverse needs, desires, capabilities, and vulnerabilities of people with dementia is essential for harnessing AI benefits while mitigating risks [18]. Thus, considering the usability of technology for dementia care is paramount for successful implementation.

### Challenges and Considerations in Technology Usability for Dementia Care

Addressing technology usability for individuals with dementia and their caregivers is recognized worldwide as a priority in national and international funding programs. Despite an increase in information and communications technologies (ICTs) for dementia care, standardized methods for evaluating ICT acceptance and usability for this demographic are lacking [19]. The technology acceptance model (TAM) and its subsequent evolutions, including the TAM 2 and the senior TAM (STAM), highlight perceived ease of use and usefulness as critical to technology adoption [20-23]. These models account for personal factors, such as cognitive abilities, and system features, such as product design and instructional support, emphasizing the adaptability of the latter to enhance usability [24,25]. Therefore, to reduce the digital divide, one of the current promising strategies is to start with product design, adopt user-centered design, and use machine learning techniques to provide timely prompts or suggestions to support and help users [24-26].

The limited adoption of digital technologies by individuals with dementia suggests that usability aspects related to product requirements and design have not been sufficiently addressed [27]. While technology usability research is advancing, there is a notable gap in literature specifically addressing the unique needs of individuals living with dementia [28,29]. This gap in clarity and consistency hampers the advancement of nursing and health science knowledge and the development of theoretical models in technology research [30]. To bridge this knowledge gap and enhance technology usability for dementia care, a concerted effort focusing on clear, tailored design principles and user-centered approaches is crucial.

### Aims

This concept analysis sought to examine the usability of technology in the context of individuals living with dementia to establish a clear definition for usability within this specific demographic.

## Methods

### Overview

Multiple sources were used to delineate the definitions and key attributes of usability specific to people living with dementia. Using the method by Walker and Avant [31], this paper offers a detailed analysis of the essential attributes of usability in the context of dementia. Their methodology uses a 7-step process that facilitates a comprehensive understanding of a concept. The process is structured as follows: (1) select a concept; (2) determine the aim of the analysis; (3) identify all uses of the concept; (4) determine the defining attributes; (5) construct model, borderline, related, and contrary cases; (6) identify antecedents and consequences of the concept; and (7) define the concept’s empirical referents.

For the literature review on technology usability for people living with dementia, works from 1984 to 2024 were searched in the electronic databases PubMed, Web of Science, and Google Scholar. The keywords applied were “technology usability” and “dementia.”

To illustrate the usability considerations of technology for individuals with mild dementia, we drew the interview data from a pilot study conducted by the third author (CB). This study involved the design and evaluation of a web-based interface tailored specifically for this population [32]. This study initially enrolled 33 dyads consisting of individuals living with dementia and their care partners. A total of 4 dyads did not complete the study for various reasons, including health-related issues and the difficulty presented by the standardized survey scales for those with dementia. Consequently, the pilot of the web application was conducted with 29 mild Alzheimer disease dementia dyads, totaling 58 older adults (29 with mild dementia and their respective care partners, with all but 1 pair being spousal dyads). Following the pilot, each dyad was interviewed regarding their experience with the web application. The participant characteristics were as follows. The average age among those living with dementia (n=29) was 70 (SD 7.06) years, ranging from 59 to 82 years, with a male majority (18/29, 62% of individuals). Most were White (28/29, 97%), and 1 participant was African American (3%). The care partners (n=29) had an average age of 68 (SD 6.73) years, ranging from 55 to 83 years, with 38% (n=11) identifying as male. Most were White (27/29, 93%), with 2 (7%) Asian American participants.

### Ethical Considerations

The interview data were drawn from a pilot study designed and evaluated by the third author (CB), focusing on a web-based interface specifically for people living with dementia [33]. The study was approved by the University of Washington Division of Human Subjects (STUDY00014226). Informed consent was obtained from all participants. The data are anonymous and have been deidentified. Each individual participant received a Visa gift card for US $150 for their time upon completion of the 3 study components.

## Results

### Concept Definition

As of now, there has been no comprehensive conceptual analysis concerning the usability of technology for individuals living with dementia. Consequently, to elucidate the use and implications of related concepts, an examination of various sources defining usability, encompassing dictionaries, organizations, and academic studies, is imperative.

#### Dictionary Definitions

*Usability* is an abstract and interdisciplinary concept. Merriam-Webster’s Online Dictionary [34] defines usability as “the quality or state of being usable: ease of use.” In the Cambridge Dictionary [35], usability is “the fact of something being easy to use, or the degree to which it is easy to use.” The Oxford English Dictionary [36] offers the following definition: “the degree to which something is able or fit to be used.” These descriptions collectively converge on a comprehensive understanding of usability as a measure of a product’s accessibility and ease of operation. Specifically, when considering individuals living with dementia, the concept of usability is tailored to the degree to which a product is easy to use or suitable for use by people living with dementia.

#### Definition From the International Organization for Standardization

The introduction section of the guidance on usability by the International Organization for Standardization [27,37] presents one of the most universally acknowledged definitions of usability. According to the International Organization for Standardization standard 9241-11, usability is defined as “the extent to which a system, product, or service can be used by specified users to achieve specified goals with effectiveness, efficiency, and satisfaction in a specified context of use” (part 11, paragraph 2) [37]. This delineation emphasizes the importance of a user-centered approach in evaluating how well a product or service facilitates the attainment of goals by individuals. Therefore, in assessing usability for people with dementia, the focus is on determining the degree to which the product or service enables effective and efficient goal achievement for this specific user group, underlining the significance of tailoring technology to meet their unique needs and enhance their quality of life.

#### Literature Definitions

While the studies reviewed did not offer explicit definitions of “usability for individuals living with dementia,” the prevailing focus within the literature revolves around the examination and assessment of technology interventions tailored for this demographic. These interventions encompass a spectrum of tools and methodologies designed to gauge acceptance, adoption, and usability among individuals living with dementia. Thus, by analyzing the content of these articles, we can derive insights into the underlying characteristics and properties associated with usability in this context. The concept of technology usability integrates essential characteristics, including ease of use, satisfaction, learnability, utility, effectiveness, efficiency, flexibility, familiarity, responsiveness, and the clarity and visibility of feedback mechanisms [38]. This comprehensive understanding of usability encompasses both objective and subjective elements, considering objective performance indicators such as actual use efficiency, effectiveness, and error rates alongside the subjective user experience and perceptions [4]. The seminal work by Meiland et al [39] classifies usability into dimensions of “user friendliness” (marked by gratification and manageability), “usefulness” (satisfying the requirements and aspirations of individuals living with dementia), and “effectiveness” (promoting independence, coping strategies, and overall well-being). These efforts to operationalize usability take into careful consideration the end users’ goals, encountered obstacles during task execution, and the selection of assistive technologies [40].

Research indicates that 40% of individuals with dementia require additional assistance and time to understand and use technological tools, including navigation through certain icons and devices. This underscores the necessity for facilitators or supervisors to aid in explaining operational steps and the use of technological intervention tools [4]. The analysis by Shultz and Hand [30] enriches our understanding of usability as the degree to which technology is perceived by users as learnable, efficient, and satisfactory. In parallel, Gibson et al [41] defined usability as the degree to which the user perceives acceptable learnability, efficiency, and satisfaction when using the technology. Gibson et al [41] advocate for a design philosophy that emphasizes personalized and adaptable support for individuals living with dementia, advocating against the use of generic technological solutions.

Further elaborating on these insights, Asghar et al [42] articulate how assistive technologies enable social interaction, health monitoring, independent mobility, and punctual medication adherence, thereby supporting daily living activities and enhancing the quality of life of those living with dementia. This perspective reiterates usability’s core definition as ease of use, efficiency, and the capacity to meet specific user needs.

The investigative effort by Miguel Cruz et al [19] into the assessment methods for the acceptance, adoption, and usability of ICTs by people living with dementia and their caregivers highlights the critical gap in standardized evaluation methodologies. This gap signals the need for further research and development to measure such technologies effectively. Hence, usability for people living with dementia hinges on the extent to which they can use specific technologies effectively, satisfactorily, and safely considering their unique requirements and cognitive limitations.

In the domain of mobile app development [43], the initiative to enhance the quality of life of those living with dementia and Alzheimer disease by creating older adult–friendly apps is paramount. This initiative directly addresses the cognitive and usability challenges faced by this demographic, aiming to make technology both accessible and beneficial.

The usability of mobile apps is fundamentally tied to 9 thematic areas: user interface design, physical considerations, screen size, interaction challenges, meeting user needs, addressing the lack of self-awareness regarding app necessities, mitigating the stigma associated with app use, overcoming technological inexperience, and emphasizing the importance of technical support [32]. These areas highlight the critical need for developing intuitive, user-friendly apps tailored to the unique challenges encountered by individuals with dementia, thereby significantly enhancing their autonomy and quality of life.

Considering the specific needs and limitations of individuals with dementia or mild cognitive impairments when designing and implementing technological interventions is critical. Through appropriate support and assistance, their user experience and the effectiveness of the intervention can be improved [4]. Usability is related to user friendliness and ease of use and learning, serving as a means for older adults and those with reduced capabilities to participate in activities and engage equally in society. Highlighting the value of involving users in technology development and clinical trials, the design of intervention studies should include people with dementia and their caregivers to understand the design features necessary to enhance usability and acceptance [9].

Kung and Chen [28] conducted a concept analysis on the usability of health promotion mobile apps, summarizing the characteristics of usability. The most common attributes of usability identified in their study included efficiency, user satisfaction, and learnability [28]. These attributes are crucial for health promotion apps, ensuring that user expectations are met, providing satisfaction, and facilitating ease of learning and use. Textbox 1 organizes the characteristics of usability for people living with dementia from the literature review. Notably, ease of use, effectiveness, efficiency, and satisfaction were frequently mentioned across various studies; these attributes are generally recognized as core characteristics of usability across various fields, not just in the context of designing for people living with dementia. Other significant attributes that repeatedly appear in the literature on usability for people living with dementia include adaptability, personalization, intuitiveness, and simplicity, suggesting a comprehensive approach to addressing the unique needs and challenges faced by people living with dementia.

**Summary of the characteristics of the definition of usability for people living with dementia.**

**Sources and attributes of usability for people living with dementia**

- Boulay et al [44], 2011: effectiveness, efficiency, and satisfaction
- Lim et al [45], 2012: intuitive, engagement, and adaptability to users’ needs
- Meiland et al [39], 2012: user friendliness, usefulness, and effectiveness
- González-Palau et al [46], 2013: effectiveness, efficiency, and satisfaction
- Yamagata et al [43], 2013: ease of use, personalization, accessibility, adaptability, and engagement
- Boger et al [47], 2015: intuitiveness, simplicity, customization, and adaptability
- Manera et al [48], 2015: effectiveness, efficiency, satisfaction, and engagement
- Martins et al [38], 2015: ease of use, satisfaction, learnability, efficacy, coherence, flexibility, and responsiveness
- Lindqvist et al [40], 2015: ease of use, effectiveness, and adaptability to users’ needs
- Shultz and Hand [30], 2015: learnability, efficiency, and satisfaction
- Ben-Sadoun et al [49], 2016: effectiveness, efficiency, and satisfaction
- Garcia-Sanjuan et al [50], 2017: effectiveness, efficiency, satisfaction, and engagement
- Meiland et al [51], 2017: effectiveness, efficiency, simplicity, intuitiveness, personalization, and engagement
- Tziraki et al [52], 2017: engagement, acceptability, and accessibility
- Asghar et al [42], 2018: effectiveness, adaptability, and satisfaction
- Holthe et al [9], 2018: effectiveness, efficiency, and satisfaction
- Gibson et al [41], 2019: adaptability, accessibility, and effectiveness
- Tuena et al [53], 2020: ease of use, intuitiveness, effectiveness, engagement, personalization, and adaptability
- Contreras-Somoza et al [4], 2021: effectiveness, efficiency, and satisfaction
- Engelsma et al [54], 2021: personalization, simplicity, clarity, engagement, and adaptability
- Koh et al [55], 2022: ease of use and engagement, customizability, and adaptability
- Neubauer et al [56], 2022: effectiveness, efficiency, simplicity, and satisfaction
- Miguel Cruz et al [19], 2023: simplicity and adaptability
- Ye et al [32], 2023: ease of use, accessibility, personalization, and effectiveness
- Zheng et al [57], 2023: effectiveness, efficiency, satisfaction, simplicity and clarity, and engagement
- Zhu et al [58], 2024: ease of use, accessibility, personalization, and engagement

#### Potentially Related but Distinct Concept of Acceptability

Acceptability of technology is the extent of the primary users’ predisposition to implement the technology in their daily activities as a result of their diverse perceptions of the product’s characteristics [59]. The primary users of technologies for people living with dementia may include individuals living with dementia or their care partners. Some studies have reported the technology acceptability of people with mild to moderate dementia and their caregivers. For example, an inaesthetic device may be interpreted as unacceptable because of its unappealing appearance and, therefore, has lower acceptability.

#### Describing Usability for People Living With Dementia From the Clinical Standpoint

Dementia progresses slowly into 3 stages: mild, moderate, and severe, which can also be called early, middle, and late stages [60]. According to the Clinical Dementia Rating (CDR), there are different stages of dementia; based on an individual’s cognitive functions, a 5-point system is used to describe each stage of dementia. A person in CDR-0 does not have dementia, and stage CDR-0.5 is considered very mild dementia or mild cognitive impairment, with slight but consistent memory problems. The expected duration of this stage is 3 to 7 years. Someone with CDR-1 has mild dementia. The average duration of this stage is 2 years, with memory loss, particularly recent events, and having trouble with the inability to perform daily tasks. In stage CDR-2, a person living with moderate dementia experiences more profound memory loss and is typically disoriented to time and place for the expected duration of 2 to 4 years. A person in stage CDR-3 is considered to have severe dementia. One might have multiple medical comorbidities, which often result in the end of functional independence. The average duration of stage CDR-3 is typically 1 to 2.5 years [60-63]. These stages help us understand how dementia may change over time and serve as a guide for the design of technology products at different stages, addressing different needs and usability.

Interactive technology refers to systems and devices that can respond to user inputs in real time, allowing for a dynamic exchange of information between the user and the technology. This domain of technology is characterized by the bidirectional provision and reception of information through actions such as language, text, movement, and touch. The key features of interactive technology include user input, real-time processing, and outputs that adjust based on user actions. Users communicate their requirements, and the technology delivers the results of their operations back to them through the interface [64,65]. Common forms of interactive technology, such as touch screens on smartphones, tablets, and information kiosks, as well as virtual reality, augmented reality, smart home devices, social media, and video games, enable users to directly interact with displayed content. Noninteractive technologies refer to products that do not require or allow for instant user input or feedback to operate. Unlike interactive technologies, which are designed to involve users in 2-way communication or interaction, noninteractive technologies, once activated or triggered, operate independently of user inputs. These technologies are often designed to perform specific tasks, display information, or execute commands without requiring ongoing user interaction. Television, smart speakers, sensors, static websites, and radio are typical examples of noninteractive technology. Operating interactive technology typically requires hand-eye coordination and precise control of the product, as well as reading or memorizing the steps or instructions on how to operate the technological product. It may even involve understanding complex user interfaces or executing multistep tasks to achieve the intended use of the product. Compared with the middle and late stages, people with mild dementia are still able to retain independence to complete many daily activities or do so with little assistance or guidance [60,66], and they are more likely to use interactive technology. For example, people living with mild dementia who attempt to have cognitive training using interactive tools to stimulate their cognitive functions might have one set of usability needs. The course of dementia is progressive, and when it enters the middle stage, people living with dementia have more profound memory loss. Their ability to cope with daily life becomes more complicated [60,66]. Due to the decline in memory functions and the ability to perform fine movements, the demand for technology products in the mid to late stages may be more often focused on noninteractive products due to usability issues.

In addition, different individuals may have different symptomology. There are different subtypes of dementia. Alzheimer disease and Lewy body dementia are the 2 most common progressive dementias. In addition to cognitive impairment, Lewy body dementia may be accompanied by muscle stiffness, hand tremors, and unsteady gait at an early stage [66]. A person with Lewy body dementia might have mobility issues that might imply the usability concern of motor activity, and someone with Alzheimer dementia might have issues with remembering instructions.

**Figure 1:**
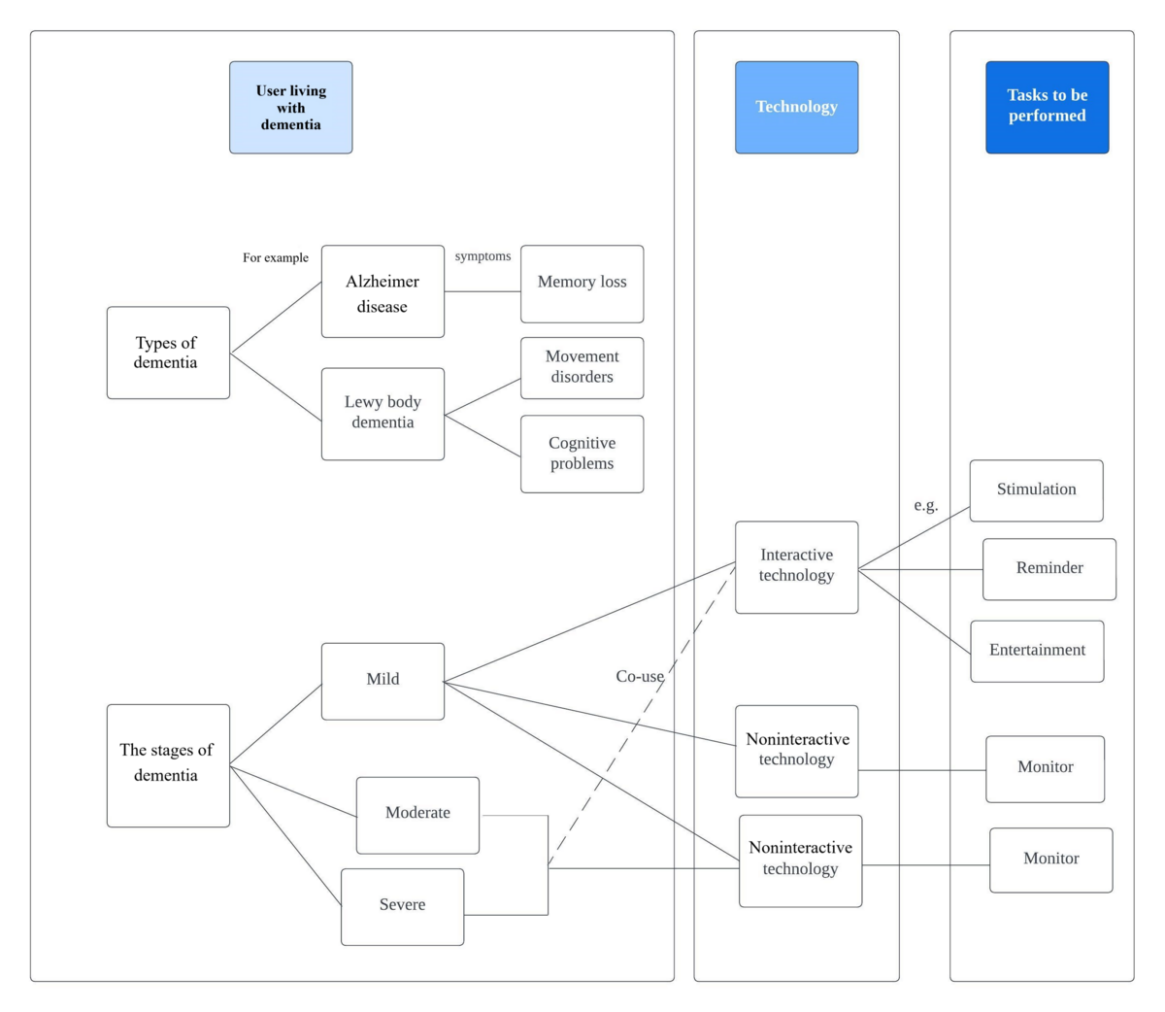
Factors affecting the use of technology for people living with dementia.

In short, from a clinical point of view, to look at the technology usability for people living with dementia, many factors require assessment, such as the stage, type, and symptoms of dementia. Furthermore, the categories of technology—interactive or noninteractive technology—should be considered based on the characteristics and needs of the disease, focusing precisely on the needs of tasks to be performed (Figure 1). In addition, people living with dementia retain certain relevant functions that are crucial for designers to consider. Despite the cognitive decline associated with dementia, individuals often maintain sensory and motor abilities provided they do not have other chronic conditions or age-related changes [67]. For designers, it is essential to recognize and leverage these retained functions when developing tools for this population. For instance, people living with dementia may be able to recall distant personal memories, which can be used in product design to evoke positive emotional and behavioral outcomes. By understanding and designing with knowledge of these maintained functions, digital tools can be better aligned with the abilities and needs of people living with dementia, thereby enhancing their engagement, enjoyment, and overall quality of life [67].

#### Definition From Interviews With People Living With Dementia

To illustrate technology usability considerations in people with mild dementia, we drew from a pilot study in which the third author designed and evaluated a web-based interface specifically for this population. In brief, the project aimed to investigate the experience of using a web application interface designed for people living with dementia and their care partners [33]. Its design complied with the criteria of the Web Content Accessibility Guidelines 2.1 at level AA, which is the standard for web accessibility developed by the World Wide Web Consortium. Components to enhance accessibility for people with sensory loss and with dementia included an audio option; screen reader compatibility; and clear, short sentences that avoided abstractions and multiple constructs. After conducting a pilot of the web application with 29 dyads of people with mild Alzheimer disease dementia and their care partners for a total of 58 older adults, each dyad was interviewed about their experience using it together. The interviewees indicated what they found positive about the web application that contributed to usability. They stated that they liked that the web application’s interface was simple, clean, straightforward, and easy to use and that there were not too many options. The buttons were specific and clearly visible, and there were not too many distractions on the screen to create confusion. Participants appreciated how age-related vision changes were considered in the design. For example, the buttons on the web application were large enough, and the texts were of a size suitable for reading. Furthermore, because they could forget the operation steps or the functions of unmarked buttons, they found the clear and explicit wayfinding directions helpful and felt that they did not risk “getting lost” in the web application. Pilot participants further appreciated personalization options, such as open-text boxes to type in explanations so that the information could be entered in a familiar way. In addition, users said that they liked the app’s summary feature, which reports the answers they chose for their future reference.

Many dyads of people with dementia and their care partners said that it was helpful to have an audio option, which read the text on the screen when selected while visually highlighting each sentence. It assisted people living with dementia in concentrating, and the care partners enjoyed not needing to read the text on the application aloud to their partners. Approximately 1 in 3 older adults experience age-related hearing loss, and their hearing sensitivity, especially high-frequency hearing, decreases gradually [68,69]. However, the ability to hear low-pitched sounds is often not affected [70]. Therefore, the web application used a male-sounding (lower) voice for reading, which can be heard more clearly by older adults. This and other age-related physiological changes are factors that must be taken into account as dementia is associated with older age [5].

Finally, in the usability-testing phase, the pilot found that it was sometimes challenging to obtain feedback from people living with dementia on the web application within a few days due to short-term memory difficulties. Some participants noted that they were unable to remember details of their experience or the web application well enough to report on it. Hence, to understand the user experience, it is necessary to find creative solutions such as soliciting real-time feedback during or immediately after using the application.

### Defining Attributes

#### Overview

Following the approach by Walker and Avant [31], after the concept of usability is defined, its essential attributes must also be defined. Particular attributes are mentioned repeatedly in the literature and can be used to distinguish this concept from those that are similar or related. The current definition and characteristics of usability are not entirely applicable to individuals with dementia. Therefore, this study synthesized a definition of usability for patients with dementia based on multiple resources, including dictionaries, a literature review, and data from interviews with people living with dementia and their caregivers. In addition to ease of use, effectiveness, acceptable learnability, and satisfaction, special attention is required for dementia symptoms and aging. Personalization becomes a crucial attribute, with adaptations based on dementia stages, subtypes, and symptoms. It emphasizes the value of simplicity, clear navigation, age-sensitive design, and personalized features, making the design of the technology or interface more specific and intuitive.

#### Constructed Cases

The application of technology to people living with dementia can be divided into the following five categories: (1) cognitive training and daily living, (2) screening, (3) health and safety monitoring, (4) leisure and socialization, and (5) navigation [71]. Selected clinical cases are listed herein to clarify and illustrate the concept of usability for people living with dementia. The case scenarios presented in this study are a combination of adapted cases from the first author’s previous clinical work and fictional scenarios, and all mentioned names are of a fictional nature. On the basis of the work by Walker and Avant [31], the exemplar case contains all the defining attributes of the concept. The borderline case possesses not all but most of the defining attributes of the concept, and the related case is related to the main defining attributes but does not contain all of them. In addition, a contrary case that has no defining attributes of this concept but is contrary to it is presented.

#### Exemplary Case

Mr Wang is a patient who was diagnosed with mild dementia 6 months ago. He experiences memory impairment, has difficulties in concentration, becomes lost easily, frequently forgets to lock the door and turn off the tap, is unable to plan trips or prepare meals as he once could, and often feels depressed. Nevertheless, he can maintain his daily life. To delay deterioration, stimulate the brain, and relieve emotional distress, his nurse provided him with a tablet computer. A total of 11 apps were preinstalled on the tablet computer based on 3 categories. The creative (art and music) app enables users to produce artwork on a blank canvas by accessing various brush types and a wide array of colors. Simple interactive games allow users to interact with virtual animals that respond to a user’s touch and gestures. Relaxation apps allow users to play relaxing background music while the tablet screen displays nature pictures [45].

After 30 minutes of demonstration, guidance, and practical exercises by the nurse, Mr Wang learned how to operate the device, including turning it on and off, charging it, adjusting the volume, navigating the home screen, and starting and exiting apps. He also received a cell phone to contact someone in case of any operation problems. After completing the standard training checklist, Mr Wang returned home with the tablet and used it for 7 days to evaluate it and the apps’ effectiveness.

The nurse invited Mr Wang’s primary caregivers to observe the status of his use of the device. Mr Wang used the tablet computer for an average of 50 minutes per day, which exceeded the recommended 30 minutes. Mr Wang expressed satisfaction with using the tablet and the apps and was willing to continue using it. According to his caregivers’ observations, Mr Wang spent more time on interactive gaming and listening to music. He spent an average of 15 minutes independently using the tablet, and he was able to independently store and charge it. Use of the tablet also enhanced his emotional stability.

#### Borderline Case

A person living with mild dementia tried to use a mobile app to buy train tickets, and their caregiver, also an older adult, assisted him in its operation (co-use). However, the app’s user interface was complicated to operate. They were finally able to buy the ticket using the app, but the process was too time intensive. This scenario involves a user living with dementia, technology, tasks to be performed (buying train tickets), and the consequences of co-use with the patient’s caregiver. The patient used the app several times afterward and became more proficient, indicating that the operation of the app is learnable for a person living with dementia. Moreover, compared with visiting the train station to purchase train tickets directly, using the app to buy tickets is more efficient. Although the purpose of using this tool was achieved, that is, it is applicable, the patient was dissatisfied with the complex and time-intensive user experience; therefore, the satisfaction attribute was not met.

#### Related Case

Mrs Smith has a diagnosis of mild dementia and lives with her family. Her daughter assisted her in downloading a diet and fitness app on her tablet computer to track nutrition, fitness, and health data. The app offered a menu and colorful photos of various diet types as well as daily exercise videos to follow. In addition, users could upload pictures and share their progress and weight control results in the app’s discussion forum, where other users could “like” and comment on the posts.

Mrs Smith enjoyed using the app for its vivid food pictures, animated exercise videos, and users sharing positive posts related to fitness progress on the forum; she felt delighted and vibrant when “liking” forum posts. For Mrs Smith, this app was useful, engaging, and a source of pleasure in her life. Hence, usefulness was present as a defining attribute. However, she could not comprehend how to record diet and health information because the operation interface was too difficult to use; the app lacked the learnability attribute. In addition, the app did not achieve the expected outcomes of its design (healthy diet and fitness), although effectiveness was not included as a defining attribute in this case. Despite this, Mrs Smith was satisfied with using this app, fulfilling the satisfaction attribute.

#### Contrary Case

A patient with severe dementia lives in a long-term care facility and is cared for by nursing staff. Because of his age and symptoms, this patient has a high risk of falling. The staff assisted in outfitting the patient with a medical alert necklace that automatically sent messages to set recipients if the patient had an emergency, such as a fall. Because this product has an automatic detection system, the patient is not required to learn how to use it, and no learnability attribute is present. The patient himself was unable to understand the function and benefits of the product as a result of the deterioration of his cognitive function; he thought something uncomfortable was stuck on his neck and often removed the necklacelike detector. Despite staff perception that it was essential to his personal safety, he was dissatisfied with it. He often removed the detector and failed to carry it, reducing the usefulness and effectiveness of the product.

#### Antecedents

The antecedents of usability for people living with dementia encompass a complex interplay among the individual living with dementia, the technology in use, the tasks to be performed, and the concept of co-use. The usability concept for people living with dementia is profoundly influenced by a complex interplay of social, environmental, and personal factors. The unique needs and capabilities of people living with dementia present various variables that affect usability, making the user living with dementia the most relevant antecedent. However, beyond the individual user, it is essential to consider the broader context of technology use. This includes the availability of space, the skills and knowledge required to operate the technology, existing support systems (such as caregivers or health care professionals), and the financial resources needed to access the technology. Furthermore, depending on the severity of dementia, patients may face greater challenges in using technological tools independently, necessitating co-use or shared use with their caregivers [72]. The concept of “co-use” underscores the importance of social dynamics in usability.

#### Consequences

**Figure 2:**
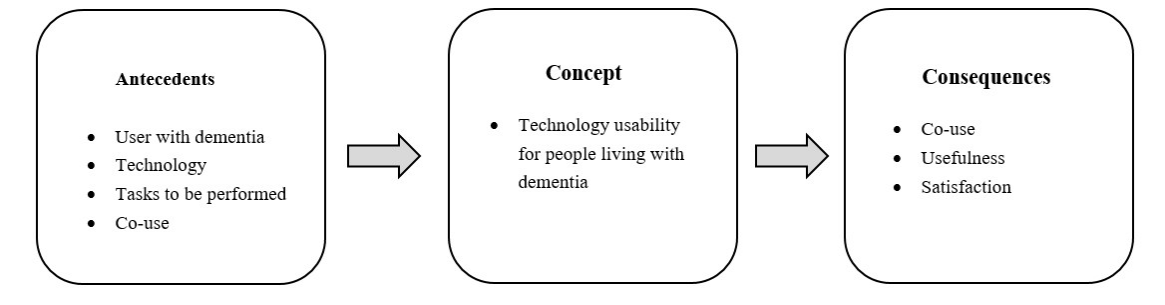
Overview of antecedents, attributes, and consequences of technology usability for people living with dementia.

The consequences of people living with dementia using technology include co-use, usefulness, and satisfaction. As progressive and irreversible cognitive impairment can affect the capability of people living with dementia to operate technological products, being able to co-use the products is one of the criteria of the consequences of the concept of technology usability. On the basis of the varying degrees of dementia severity, patients who exhibit more pronounced symptoms often encounter greater challenges in independently using technological tools. This situation necessitates a collaborative approach where co-use or shared use with caregivers becomes essential. The need for such support intensifies as the severity of the dementia increases, highlighting the importance of adaptable technological solutions and the involvement of caregivers in the use process to ensure effective and safe interaction with these tools [72]. In addition, the research by Astell et al [73] on the use of interactive multimedia touch screen systems for people living with dementia found that interacting with caregivers through touch screen tablets was intriguing and engaging for people living with dementia. These consequences are similar to the attributes that define usability. Nevertheless, the consequence of usability is the result of the direct evaluation of specific technological applications by people living with dementia, including co-use, perceptions of usefulness, and satisfaction [30] (Figure 2).

### Empirical Referents

#### Overview

A deeper understanding of the concept of technology usability for people living with dementia can be used as a reference index for clinical practice in assisting those living with dementia in locating suitable technological interventions that promote a healthy and comfortable life. Although the exact definitions and testing of usability vary for different products, their effectiveness is reflected in the actual user operation, that is, whether the device achieves its main goal and is satisfactory for the users (people living with dementia). When testing usability, certain elements must be considered, such as the target users; their learning abilities, desires, and needs; the environment in which they operate; and what advantages the product offers [73]. Usability evaluation methods include quantitative methods such as questionnaires and task completion and qualitative methods such as the “think-aloud” protocol, focus groups, and interviews [74].

#### Think-Aloud Methods

Among the evaluation methods, the think-aloud (or thinking-aloud) protocol is the most commonly applied qualitative method used to collect data in usability testing. Users are required to speak their thoughts, actions, and perceptions aloud while operating a product, with observers objectively recording the user’s speech verbatim without interpreting their behaviors or statements [75]. Customarily, the test is filmed for developer review; developers observe the user process of operating the product as well as users’ response to ascertain their experience of the application [76].

#### System Usability Scale

This is the most widely used and validated questionnaire and comprises a 10-item Likert scale. This simple, standardized questionnaire is advantageous for its objectivity, generality, repeatability, and quantifiability. The questions focus on frequency of use, complexity of operation, consistency, degree of preference, and achievability of the product goal [77]. However, because the think-aloud protocol has higher demands of cognitive load for people living with dementia [76] and the System Usability Scale, a posttest tool, cannot reflect how people living with dementia interact with technology products timely, there is currently no clear, standardized method to assess usability for people living with dementia. Hence, there is still a higher need to design more consistent and reliable evaluation instruments for people living with dementia [19,76].

#### Observation and Logging

Other primary methods for evaluating usability for people living with dementia are observational methods and logging, which present more objective data, with observations enhanced by video recordings for more reliable results [32,76]. However, observer bias, the presence and perceptions of the observer, can influence both the observer’s interpretations of what is seen and possibly the behavior of the participants being observed.

Therefore, it is advised to combine both objective and subjective measures for a comprehensive understanding of usability needs [78,79] and consider the aims of the research to select the proper measuring tool.

We suggest that, when considering usability for people living with dementia, it is necessary to understand the characteristics of users. First, the symptoms and needs of dementia at each stage, the designing purpose, and the tasks to be performed should be understood. Furthermore, dementia mostly affects older adults, so corresponding designs should take into account diverse physical needs, potential sensory loss, and other age-related changes. In addition, primary caregivers spend a lot of time and energy accompanying and caring for people living with dementia. Their firsthand observations, insights, and opinions can be informative when testing products, particularly for technology products designed for co-use. As the disease progresses, changes in condition and symptoms need to be considered. Another suggestion is to design diversified and multifunctional technological products to provide more inclusive and flexible choices and adjust software features to meet individual needs. Product function classification could be upgradable or downgradable as the condition of the user changes, and these functions could be selected by the user.

Furthermore, Sebastian et al [80] have raised a question about the potential of rhetoric in enhancing the adoption of AI; their study results suggest that the adoption of strategic communication techniques (ethos, pathos, and logos) can significantly impact people’s willingness to accept AI technologies. Our conceptual analysis also acknowledges the importance of effective communication strategies in facilitating technology adoption. This insight is particularly significant in technology adoption among individuals living with dementia, where trust in and understanding of technology plays a crucial role. Therefore, future research could explore how customized communication strategies based on rhetorical principles can address the concerns and needs of people living with dementia and their caregivers.

## Discussion

### Clarifying and Validating the Concept of Usability for Individuals With Dementia

To our knowledge, this study represents the first concept analysis specifically focused on usability for individuals with dementia. This paper integrates existing literature and combines empirical data obtained from interviews to relate the concept to specific real-world situations. It offers insights into usability within the context of dementia, covering its practical significance and applications as well as directions for future research.

The concept of usability, particularly for individuals living with dementia, demands a nuanced understanding that accounts for the rapid advancements in technology and its increasing application in supporting these individuals and their caregivers. The specific needs of individuals with dementia, tailored to accommodate common symptoms and optimize their capabilities, underline the critical need for a precise and comprehensive definition of technology usability within this context.

Usability is acknowledged as a multidimensional construct that becomes tangible only when technology is actively used by individuals. The variability of the usability definition, contingent on the context and specific application field, presents a challenge in achieving a unified conceptualization [19,81]. This challenge is further compounded for individuals living with dementia due to the complex interplay between the severity and subtype of dementia and specific technological requirements, including the necessary level of interactivity, technical characteristics, and intended tasks.

Given the diverse severities and progression rates of dementia, individuals experience varying needs across different stages of the condition, necessitating distinct technological interventions and, consequently, different usability requirements at each phase. Therefore, as delineated in Textbox 1, beyond the standard usability attributes—ease of use, effectiveness, efficiency, and satisfaction—additional attributes pertinent to individuals living with dementia include intuitiveness, simplicity, personalization, and adaptability.

Intuitiveness refers to the ease with which users can understand and interact with technology or an interface without previous instruction. When technology is intuitive, individuals living with dementia are more likely to use it independently, fostering a sense of autonomy and confidence. Simplicity, on the other hand, emphasizes clean design and straightforward functionalities that avoid overwhelming the user. It ensures that the cognitive load is minimized, which is particularly important given the cognitive challenges associated with dementia. Together, these attributes create a user-friendly environment that supports the engagement and sustained use of technology by minimizing frustration and maximizing ease of use.

Personalization significantly enhances usability and the overall user experience. Although not a traditional usability attribute, it reflects an understanding of the variability in dementia symptoms and stages, requiring solutions tailored to the individual’s changing needs. This approach specifically addresses the challenges associated with various stages and types of dementia. Adaptability complements this concept by ensuring that technology can adjust to the user’s changing condition over time, particularly as dementia progresses.

### Exploring the Integration With Existing Theoretical Frameworks

The TAM posits that perceived ease of use and perceived usefulness are the 2 main factors affecting one’s beliefs, intentions, and attitudes toward using novel technology [20,21]. These factors become even more significant in the context of technology development for patients with dementia, where cognitive impairments necessitate a more detailed and specific consideration of usability. This underscores the need to optimize technology products based on the specific abilities of users. Venkatesh and Davis [22] developed the TAM 2, an expansion of the original TAM, which highlights the importance of social influence and cognitive instrumental processes in technology acceptance and use, including aspects such as subjective norms, voluntariness, and individual experiences. These factors may play a crucial role in determining whether patients with dementia are willing to adopt certain technologies. However, for individuals with dementia, the usability of technology products is particularly critical as they may encounter specific challenges in cognition and perception that are not as pronounced in the general user groups highlighted by the TAM and TAM 2. This means that, for this unique population, perceived ease of use and perceived usefulness remain critical factors but there is also a need to further consider how products can be designed and supported to meet their specific needs.

The STAM, built on the foundation of the TAM and specifically designed to address the needs of older adults, including those with dementia, focuses on technology adoption among older adults [23]. The STAM maintains that perceived usefulness and ease of use, key factors in technology adoption across different age groups, are especially critical for older adults. Moreover, it adds that gerontechnology self-efficacy, gerontechnology anxiety, and facilitating conditions, which are age-related health and ability characteristics more relevant to older adults, are vital in designing technology products for patients with dementia. These considerations emphasize the necessity of considering their cognitive limitations and technology use experience as well as how to minimize barriers to technology use through facilitating conditions.

Therefore, based on the usability for people living with dementia, it is suggested to develop or expand the existing TAMs and theories by (1) incorporating specific factors related to cognitive impairments, adding variables related to cognitive load, information processing speed, and memory retention capabilities; (2) considering contextual and environmental factors such as the home environment, support from care institutions, and the impact of social and cultural backgrounds on technology acceptance and use; (3) emphasizing the importance of user experience design, especially in terms of how design can reduce anxiety and boost confidence among users with dementia, thereby promoting technology acceptance [4]; and (4) focusing on personalized and adaptive design for patients with dementia, expanding the model to include principles of adaptability and personalization according to individual differences, considering the varying needs of people at different stages of dementia [32].

### Recommendations for Standardized Usability Testing for People Living With Dementia

We recommend that, when developing a standardized usability test for people with dementia, it is essential to integrate several elements, particularly considering the progression and variability of symptoms. In addressing the usability attributes relevant to people living with dementia, the use of adaptive testing methods is advised. These methods involve designing usability tests that can be customized for different stages of dementia, adjusting protocols based on the cognitive, physical, and emotional states of participants. Due to the progressive nature of dementia, regular re-evaluation is necessary. Immediate feedback after testing is important due to short-term memory difficulties in people living with dementia. To prevent attention deficits or fatigue in people living with mild to moderate stages of dementia, testing durations should be limited as participants might become distracted and fatigued after 30 to 35 minutes of continuous dialogue [33]. Inclusive design principles must consider a broad range of abilities and limitations, ensuring test accessibility for various dementia stages. Finally, collaboration with experts such as health care professionals and dementia specialists is vital for deeper insights into the needs and challenges of people living with dementia.

### Potential Frameworks for Usability Assessment for People Living With Dementia

Furthermore, given the unique needs and challenges faced by people living with dementia, addressing the need for a standardized usability assessment specifically tailored to them is important. Some potential frameworks or methods that could be considered or developed for this purpose include, first, human-centered design approach. In this approach, the focus is on involving people living with dementia and their caregivers in the design process by adopting a human-centered design for the intervention, involving an interactive development process that focuses on the users and their needs and requirements [82]. This could include interviews, focus groups, and usability testing sessions with prototypes [74]. Participants are the ones who understand their needs best; using this method, they will also be the designers, involved in the designing phase. It is crucial to incorporate their feedback into the development process to tailor the product to meet their specific capabilities, needs, and preferences. The second potential method is contextual design and observational studies. This method consists of observing people living with dementia interacting with technology in their usual environment. It helps understand how they use technology, what challenges they face, and what aspects of the technology are most beneficial or problematic for them [83]. The third method is heuristic evaluation, a method in which usability experts analyze a product using a set of 10 heuristics [84] and that can be adapted for people living with dementia by involving dementia specialists and usability professionals [85]. The fourth method is longitudinal studies. As dementia is a progressive condition, longitudinal studies can be valuable in assessing how usability needs change over time and how well technology adapts to these changing needs.

### Limitations and Future Research Directions

Despite obtaining rich information through interview data, this research still has limitations. One of the limitations is the high homogeneity in terms of the participants’ race and ethnicity, which could potentially impact the generalizability of the research findings. In addition, it is suggested that future research collect data on participants’ educational background and socioeconomic status as these factors may be related to their willingness and ability to use technology products.

### Conclusions

This study, through a concept analysis that included interviews with people living with dementia and their caregivers, clarified the definition and attributes of usability tailored for this population. A precise definition of usability in this context is crucial to guide future research and practical applications. This study stresses the importance of considering dementia-specific aspects such as the symptoms and aging process. Customization, guided by the disease’s stages, subtypes, and symptoms, is emphasized as critical. Therefore, the design of interventions for individuals with dementia should prioritize simplicity, clear navigation, age-appropriate esthetics, and personalization to enhance specificity and intuitiveness. Furthermore, it is necessary to consider the antecedents, attributes, and consequences of technology usability for this demographic. Adopting a comprehensive approach is pivotal for developing technology solutions that are finely attuned to the unique needs of people living with dementia, fostering a nuanced understanding of usability in this context.

## Abbreviations

AI: artificial intelligence
CDR: Clinical Dementia Rating
ICT: information and communications technology
STAM: senior technology acceptance model
TAM: technology acceptance model

## Data Availability

The data sets analyzed during this study are not publicly available due to the reuse restrictions outlined in the consent form.
